# Elevated lipoprotein(a) and adverse outcomes in advanced coronary artery calcification: An intravascular ultrasound study

**DOI:** 10.1016/j.ijcrp.2026.200606

**Published:** 2026-02-20

**Authors:** Shaowu Xiao, Mengya Zeng, Junru He, Dabao Xiao, Yuewu Chen

**Affiliations:** a Department of Cardiovascular Internal Medicine, The Second Affiliated Hospital of Hainan Medical University, Haikou, 570103, China; b Hainan Medical University, Haikou, 571199, China

**Keywords:** Lipoprotein(a), Coronary artery calcification, Intravascular ultrasound, Major adverse cardiovascular events, Constrictive remodeling

## Abstract

**Background:**

Coronary artery calcification (CAC) signifies advanced atherosclerosis and portends increased cardiovascular risk. Lipoprotein(a) [Lp(a)] is a causal risk factor for atherosclerosis; however, its association with in vivo lesion morphology and clinical outcomes in patients with symptomatic, advanced CAC remains incompletely characterized.

**Objective:**

This study aimed to investigate the association between elevated Lp(a) levels and both in vivo lesion morphology and clinical outcomes in this high-risk population.

**Methods:**

In this retrospective cohort, 292 patients with intravascular ultrasound(IVUS)-confirmed CAC were stratified into elevated (≥50 mg/dL,n = 77) or low (<50 mg/dL,n = 215) Lp(a) groups. The primary endpoint was major adverse cardiovascular events (MACEs). Associations were assessed via multivariable Cox models adjusted for clinical covariates.

**Results:**

Patients in the elevated Lp(a) group presented a greater incidence of aortic valve calcification (p < 0.001). IVUS revealed constrictive remodeling with a smaller lumen and vessel dimensions. During a median follow-up of 17.2 months, the elevated Lp(a) cohort had a significantly higher MACE rate (37.7% vs. 15.8%; adjusted hazard ratio [aHR] 2.60, 95% CI 1.55–4.35, p < 0.001). Elevated Lp(a) independently predicted increased risks of ischemic stroke (aHR 7.14) and in-stent restenosis (aHR 2.78).

**Conclusion:**

In symptomatic patients with IVUS-confirmed CAC, elevated Lp(a) identifies a high-risk phenotype characterized by constrictive vascular remodeling and a markedly increased risk of MACEs, driven particularly by ischemic stroke and in-stent restenosis. These findings support the integration of routine Lp(a) testing into the risk stratification of patients with severe CAC, thereby identifying a precise high-risk phenotype that warrants intensified monitoring and represents an ideal target for emerging Lp(a)-lowering therapies.

## Highlights

• Elevated Lp(a) (≥50 mg/dL) identifies high-risk patients with symptomatic, IVUS-confirmed coronary artery calcification.
• High Lp(a) is associated with a distinct constrictive vascular remodeling phenotype on IVUS.
• Elevated Lp(a) confers a >7-fold higher risk of ischemic stroke in this advanced CAC cohort.
• In-stent restenosis risk is nearly tripled in patients with elevated Lp(a) and severe calcification.
• Aortic valve calcification is over 4 times more prevalent in patients with elevated Lp(a).

## Graphical abstract

**Figure:**
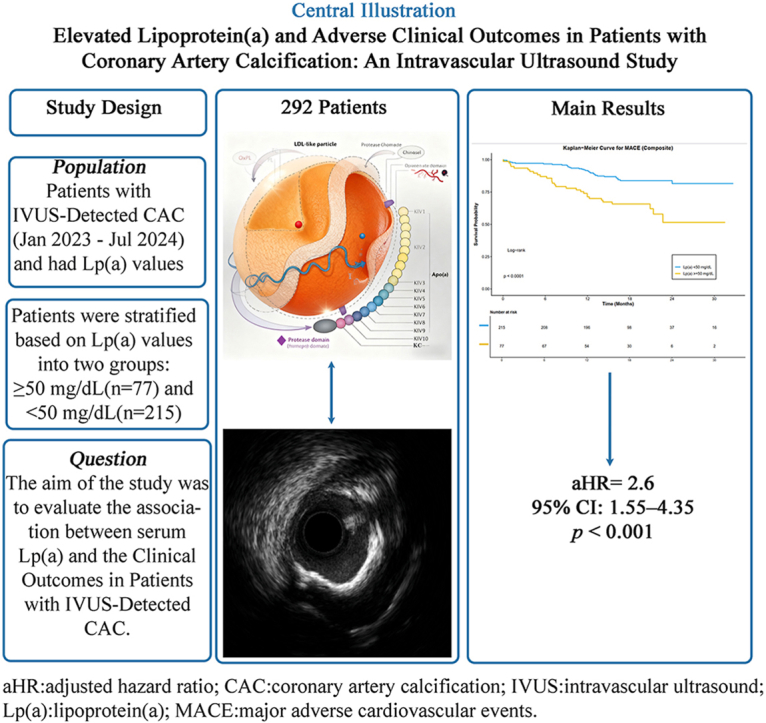

## 1 Introduction

Coronary artery calcification (CAC), characterized by hydroxyapatite crystal deposition in the intima, is a recognized indicator of advanced atherosclerosis [1]. The burden of CAC strongly correlates with the total plaque load and independently forecasts the risk of major adverse cardiovascular events (MACEs) [2]. However, even with optimal medical therapy and contemporary percutaneous coronary intervention (PCI), significant CAC continues to portend substantial risk, being closely linked to recurrent ischemia, stent failure, and increased mortality [3]. This persistent vulnerability in patients with advanced, symptomatic CAC underscores the need to identify modifiable risk drivers, such as lipoprotein(a) [Lp(a)].

Lp(a) is a key residual risk factor in cardiovascular disease that operates through proatherogenic, proinflammatory, and prothrombotic mechanisms [4]. Elevated Lp(a) levels have been consistently validated as causal, independent risk factors for coronary artery disease (CAD), aortic stenosis, and ischemic stroke [[4], [5], [6]].

Critical knowledge gaps persist regarding the interplay between Lp(a) and the in vivo architectural features of advanced CAC. Large-scale epidemiological studies (e.g., MESA, UK Biobank) have established Lp(a) as a risk factor in general and primary prevention populations [[7], [8], [9]]. However, a critical gap exists in understanding its role in advanced, symptomatic atherosclerotic disease requiring intervention. We therefore conducted this study to test the hypothesis that in symptomatic patients with IVUS-verified CACs in target lesions—a precisely defined, high-risk phenotype—elevated Lp(a) levels are associated with distinct vascular remodeling and predict adverse clinical outcomes. Unlike population-based CT studies that quantify the global calcific burden, our IVUS-based approach provides high-resolution, lesion-specific characterization of calcium and vessel architecture, offering unique mechanistic insights into the pathophysiology of advanced, intervention-requiring calcific plaques.

To bridge this gap, we conducted this IVUS-based study to test the hypothesis that elevated Lp(a) not only identifies patients with CAC at extreme risk for stroke and stent failure but also delineates a specific ‘constrictive remodeling’ phenotype. This phenotype may reflect a pervasive Lp(a)-driven pathobiology that persists despite standard care, thus representing a high-priority target for emerging Lp(a)-lowering therapies.

## 2 Methods

### 2.1 Study design and population

This single-center, retrospective cohort study was conducted at the Department of Cardiovascular Internal Medicine, The Second Affiliated Hospital of Hainan Medical University. This study was reported in accordance with the Strengthening the Reporting of Observational Studies in Epidemiology (STROBE) statement for cohort studies.

Between January 2023 and July 2024, we consecutively screened 762 patients who had undergone IVUS examination before PCI. The key inclusion criterion were: (1) age ≥18 years; (2) the presence of IVUS-confirmed CAC, defined as a bright, high-echoic area with acoustic shadowing, in at least one target lesion planned for evaluation or intervention.

In line with contemporary interventional practice, IVUS was performed on clinically indicated culprit or target lesions only and was not used as a systematic screening tool for total coronary calcium burden. Consequently, our cohort represents patients with ‘IVUS-confirmed CAC in a target lesion’—a phenotype of symptomatic, locally advanced calcific disease.

The application of the exclusion criteria led to the exclusion of 447 patients without CACs in the imaged lesion, 2 with poor-quality IVUS images, 4 with missing identification information, 12 duplicate records, and 5 with missing Lp(a) data. Consequently, the final analysis cohort comprised 292 patients.

**Fig. 1:**
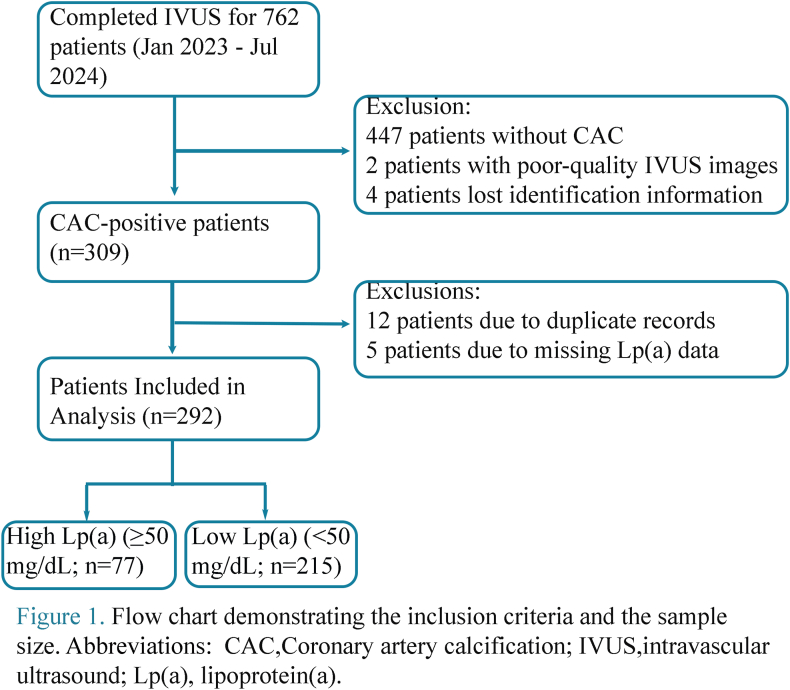
Flow chart demonstrating the inclusion criteria and the sample size. CAC, Coronary artery calcification; IVUS, intravascular ultrasound; Lp(a), lipoprotein(a).

On the basis of plasma Lp(a) levels, the study population was categorized into a high-concentration group (Lp(a) ≥50 mg/dL, n = 77) and a low-concentration group (Lp(a) < 50 mg/dL, n = 215). The patient enrollment process is detailed in Fig. 1.

Sex and gender considerations: As a retrospective analysis, our study considered the variable of biological sex as a key demographic factor. The interaction effect between sex and Lp(a) group was formally tested by including an interaction term in the multivariable Cox model. This study considered biological sex as an important demographic and biological variable. Sex-disaggregated analyses were performed, and reporting followed the Sex and Gender Equity in Research (SAGER) guidelines where applicable.

### 2.2 Sample size and power analysis

As a retrospective observational study analyzing all consecutive eligible patients, a prospective sample size calculation was not performed. A post hoc power analysis confirmed >90% power to detect the observed hazard ratio for the primary endpoint (Supplementary Fig. 1). The study included all consecutive eligible patients during the study period, thereby minimizing selection bias and maximizing the representativeness of the target clinical population.

### 2.3 Data collection

Study covariates, including established CAD risk factors, were extracted from institutional electronic medical records. The data collected included demographics, laboratory values, PCI procedural characteristics, IVUS parameters, and discharge medications. The following variables were rigorously ascertained: age, sex, body mass index, blood pressure, smoking status, and comorbidities (including hypertension, diabetes, dyslipidemia, and chronic kidney disease). Clinical histories of prior myocardial infarction (MI) and PCI were also recorded. The laboratory parameters included metabolic profiles, renal function, lipid levels [including Lp(a)], hematological parameters, and biomarkers of inflammation, myocardial injury, and cardiac function. To ensure temporal relevance, we used the Lp(a) measurement taken closest to the time of the index procedure. Sex-disaggregated data were systematically collected for baseline characteristics to enable sex-based analyses.

**Fig. 2:**
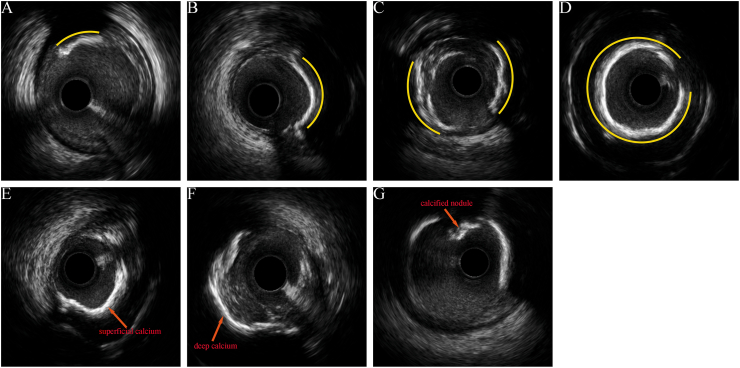
Types of coronary artery calcification on intravascular ultrasound. A, Grade 1 (0-90°); B, Grade 2 (91-180°); C, Grade 3 (181-270°); D, Grade 4 (271-360°); E, Superficial calcium; F, Deep calcium; G, Calcified nodule. The yellow line outlines the calcific arc. Figures from authors' organization. (For interpretation of the references to colour in this figure legend, the reader is referred to the Web version of this article.)

Serum Lp(a) concentrations were measured via an Lp(a) Assay Kit (Latex-Enhanced Immunoturbidimetric Method; Mindray Biomedical Electronics Co., Ltd., Shenzhen, China). The assay's interassay and intra-assay coefficients of variation were <5%. The assay calibrators were traceable to the IFCC reference material SRM 2B. An Lp(a) level of ≥50 mg/dL was used as the cutoff to define elevated risk [4,10]. Automated IVUS pullback was performed at 1 mm/s (30 frames/s) via an iLab™ Polaris Multi-Modality Guidance System (Boston Scientific). Offline analysis was conducted via proprietary Image Viewer software (version 1.6). Offline IVUS analyses were performed by two experienced investigators blinded to the patients' clinical data and Lp(a) levels. Blinding was achieved by using anonymized IVUS image files with all patient identifiers removed prior to analysis. Representative IVUS images illustrating calcification [11] are provided in Fig. 2.

### 2.4 Study endpoints and follow-up

Patients were followed from the index procedure until the occurrence of MACEs, death, or September 30, 2025 (the data lock point), whichever came first. MACEs, the primary endpoint, were defined as a composite of cardiovascular death, nonfatal MI, ischemic stroke, target vessel revascularization (TVR), hospitalization for heart failure, or in-stent restenosis (ISR). The secondary endpoints assessed were the individual occurrences of these events.

Endpoint events were ascertained retrospectively by reviewing electronic medical records for readmission data, supplemented by structured telephone follow-ups. To ensure objectivity, all potential events were adjudicated by an independent clinical events committee blinded to patient group assignments. The endpoint definitions were as follows.

1. Cardiovascular death: death due to acute MI, sudden cardiac death, heart failure, stroke, cardiovascular complications, cardiovascular hemorrhage, or other established cardiovascular causes [12].
2. Nonfatal MI: Diagnosis according to the Fourth Universal Definition of MI [13].
3. Ischemic stroke: A new focal neurological deficit of cerebrovascular origin lasting ≥24 h or leading to death, confirmed by neurologist assessment and neuroimaging (CT/MRI) [14].
4. TVR: Percutaneous intervention or surgical bypass of any segment within the entire major coronary vessel previously treated should be repeated.
5. Hospitalization for heart failure: An inpatient admission or urgent visit characterized by the requirement for intravenous diuretics due to new or worsening heart failure, with supporting objective evidence (e.g., pulmonary edema on X-ray, echocardiographic dysfunction, or elevated natriuretic peptides) [15].
6. ISR: Luminal renarrowing ≥50% within the stent or its 5-mm margins on follow-up angiography, associated with recurrent symptoms or objective evidence of ischemia [16].

### 2.5 Statistical analysis

Continuous variables are described as the mean ± standard deviation or median [interquartile range] on the basis of their distribution, which was evaluated via the Shapiro‒Wilk test and histogram inspection. Categorical variables are reported as frequencies (percentages). Group differences (high vs. low Lp(a)) were assessed with Student's *t*-test, the Mann-Whitney *U* test, or the chi-square test, as appropriate for the variable type and distribution.

Kaplan‒Meier curves were plotted to visualize cumulative event rates, and the log-rank test was used for group comparisons. Univariable and multivariable Cox proportional hazards models were employed to evaluate the effect of Lp(a) on outcomes. Covariates for the multivariable model were selected a priori on the basis of established clinical relevance. The multivariable model included adjustments for age, sex, smoking, hypertension, hyperlipidemia, diabetes, history of MI, history of PCI, and glomerular filtration rate (GFR). The interaction effect between sex and Lp(a) group was formally tested by introducing an interaction term into the multivariable Cox model. A two-sided interaction p value < 0.10 was considered statistically significant for effect modification by sex.

Given that missing data are minimal (<5%) and are assumed to be missing completely at random, a complete-case analysis was applied. A two-sided p value < 0.05 was considered statistically significant. All computations were carried out in R (version 4.5.1).

## 3 Results

### 3.1 Baseline characteristics

Table 1 presents the baseline characteristics of the 292 enrolled patients, stratified by an Lp(a) cutoff of 50 mg/dL. The cohort had a mean age of 66.3 ± 10.6 years, and 73.6% were male. Overall, 77 patients (26.4%) had elevated Lp(a) levels (≥50 mg/dL). The frequency distribution of the Lp(a) concentration is shown in Supplementary Fig. 2.

**Table 1:** Baseline characteristics.

	Overall	Lp(a) < 50 mg/dL	Lp(a)≥50 mg/dL	P value
	(n = 292)	N = 215(73.6%)	N = 77(26.4%)	
Patient demographics				
Age, years	66.3 ± 10.6	66.2 ± 10.8	66.3 ± 10.0	0.954
Gender,n(%)				0.587
Male	215(73.6%)	156(72.6%)	59(76.6%)	
Female	77(26.4%)	59(27.4%)	18(23.4%)	
Body mass index, kg/m2	23.7(21.7,25.8)	23.8(21.7,26.1)	23.7(21.8,25.9)	0.873
SBP,mmHg	135(119,155)	134(120,155)	139(119,158)	0.438
DBP,mmHg	78(71,88)	78(72,88)	77(69,87)	0.455
Medical history,n(%)				
Smoking	80(27.4%)	62(28.8%)	18(23.4%)	0.440
Hypertension	191(65.4%)	135(62.8%)	56(72.7%)	0.152
Dyslipidemia	55(18.8%)	45(20.9%)	10(13.0%)	0.174
Diabetes mellitus	113(38.7%)	81(37.7%)	32(41.6%)	0.643
Chronic kidney disease	20(6.8%)	11(5.1%)	9(11.7%)	0.090
Prior myocardial infarction	58(19.9%)	44(20.5%)	14(18.2%)	0.791
Prior PCI	90(30.8%)	61(28.4%)	29(37.7%)	0.170
Laboratory				
Glucose, mmol/L	6.3(5.3,8.7)	6.1(5.0,8.0)	6.7(5.6,10.6)	0.013
Hemoglobin A1c,%	6.3(6.0,7.4)	6.3(6.0,7.3)	6.5(6.0,7.5)	0.664
Serum creatinine,μmol/L	82.5(66.0,100.3)	80.0(65.5,95.5)	85.0(66.0,114.0)	0.094
GFR,mL/min/1.73 m^2^	83.5(67.4,97.0)	84.0(69.5,97.0)	80.5(61.0,96.0)	0.140
Triglycerides, mmol/L	1.37(0.97,1.98)	1.36(0.94,1.98)	1.40(1.11,2.05)	0.425
Total cholesterol, mmol/L	4.64(3.66,5.45)	4.59(3.66,5.47)	4.70(3.71,5.42)	0.778
LDL-C,mmol/L	2.80(2.05,3.55)	2.80(2.05,3.57)	2.86(2.04,3.49)	0.813
HDL-C,mmol/L	1.02(0.85,1.23)	1.02(0.84,1.23)	1.04(0.87,1.19)	0.531
Lp(a), mg/dL	19.6(7.6,50.7)	13.2(5.9,23.3)	63.0(55.3,84.6)	<0.001
CK-MB,U/L	14(9,24)	14(9,23)	15(10,26)	0.349
Cardiac troponin I,ng/ml	0.01(0.01,1.32)	0.01(0.01,1.06)	0.02(0.01,1.79)	0.041
NT-proBNP,pg/mL	328(95,1621)	275(84,1113)	947(145,2760)	0.008
White blood cell, × 10^9^/L	7.7(6.2,9.9)	7.7(6.2,9.9)	7.5(6.4,10.3)	0.889
Neutrophil, × 10^9^/L	4.7(3.7,7.4)	4.7(3.6,7.3)	4.9(3.9,8.0)	0.395
Monocyte, × 10^9^/L	0.51(0.41,0.69)	0.50(0.41,0.68)	0.53(0.42,0.72)	0.644
Platelet, × 10^9^/L	232(197,273)	234(200,273)	230(188,280)	0.665
hs-CRP,mg/L	3.98(1.31,12.25)	3.48(1.13,10.68)	4.56(2.17,19.50)	0.041
Ejection fraction,%	60(51,65)	61(54,65)	56(47,64)	0.043
Clinical presentation,n(%)				0.263
STEMI	40(13.7%)	31(14.4%)	9(11.7%)	0.686
NSTEMI	54(18.5%)	36(16.7%)	18(23.4%)	0.265
Unstable angina	142(48.6%)	102(47.4%)	40(52.0%)	0.585
Stable angina	56(19.2%)	46(21.4%)	10(13.0%)	0.150
Comorbidities,n(%)				
Aortic valve calcification	30(10.3%)	12(5.6%)	18(23.4%)	<0.001
NAVC	14(4.8%)	9(4.2%)	5(6.5%)	0.615
Aortic Stenosis	4(1.4%)	3(1.4%)	1(1.3%)	0.999
Discharge Medications,n(%)				
ACEI/ARB	186(63.7%)	138(64.2%)	48(62.3%)	0.880
Beta-blocker	206(70.5%)	149(69.3%)	57(74.0%)	0.526
Statins	285(97.6%)	211(98.1%)	74(96.1%)	0.570
Dual antiplatelet therapy	268(91.8%)	195(90.7%)	73(94.8%)	0.377
Aspirin	275(94.2%)	202(94.0%)	73(94.8%)	0.999
Clopidogrel	175(59.9%)	127(59.1%)	48(62.3%)	0.714
Ticagrelor	112(38.4%)	83(38.6%)	29(37.7%)	0.993

ACE-I/ARBs, angiotensin-converting enzyme inhibitors/angiotensin receptor blockers; CK-MB,creatine kinase myocardial band; DBP,diastolic blood pressure; GFR,glomerular filtration rate; HDL-C,high-density lipoprotein; hs-CRP, high-sensitivity C-reactive protein; LDL-C, low-density lipoprotein; Lp(a), lipoprotein(a); NAVC, Nonaortic cardiac valve calcification; NSTEMI,non-ST segment elevation acute myocardial infarction; NT-proBNP,N-terminal pro-B type natriuretic peptide; PCI,percutaneous coronary intervention; SBP, systolic blood pressure; STEMI,ST segment elevation acute myocardial infarction.

The two groups were well balanced with respect to demographics and most of their clinical histories. However, the high Lp(a) group tended to have a greater incidence of chronic kidney disease (11.7% vs. 5.1%, p = 0.090) and prior PCI (37.7% vs. 28.4%, p = 0.170), although these differences did not reach statistical significance.

Significant differences were observed in the laboratory profiles. Compared with patients in the low Lp(a) group, patients in the high Lp(a) group presented elevated levels of random glucose (6.7 [5.6,10.6] vs. 6.1 [5.0,8.0] mmol/L, p = 0.013), cardiac troponin I (0.02 [0.01,1.79] vs. 0.01 [0.01,1.06] ng/mL, p = 0.041), NT-proBNP (947 [145,2760] vs. 275 [84,1113] pg/mL, p = 0.008), and hs-CRP (4.56 [2.17,19.50] vs. 3.48 [1.13,10.68] mg/L, p = 0.041), alongside a lower left ventricular ejection fraction (56 [47,64]% vs. 61 [54,65]%, p = 0.043). Lp(a) stratification was confirmed by markedly different levels between the groups (63.0 [55.3, 84.6] vs. 13.2 [5.9, 23.3] mg/dL, p < 0.001). No statistically significant differences were found in other lipid parameters, including LDL-C, HDL-C, and triglycerides.

The high Lp(a) group had a significantly greater burden of aortic valve calcification (23.4% vs. 5.6%, p < 0.001), despite similar clinical presentations and nearly identical discharge medication regimens, including statin use (>96%).

### 3.2 IVUS characteristics and PCI features

Table 2 summarizes the IVUS-defined characteristics of the CAC and PCI procedures.

**Table 2:** IVUS-defined characteristics of CAC in PCI.

	Overall	Lp(a) < 50 mg/dL	Lp(a)≥50 mg/dL	P value
	(n = 292)	N = 215(73.6%)	N = 77(26.4%)	
Calcification Site,n(%)				
Left main	60(20.5%)	48(22.3%)	12(15.6%)	0.275
Left anterior descending artery	215(73.6%)	158(73.5%)	57(74.0%)	0.999
Left circumflex artery	38(13.0%)	31(14.4%)	7(9.1%)	0.320
Right coronary artery	70(24.0%)	51(23.7%)	19(24.7%)	0.990
CAC Characterization,n(%)				
Depth-based classification				
Superficial calcium	286(97.9%)	210(97.7%)	76(98.7%)	0.939
Deep calcium	62(21.2%)	45(20.9%)	17(22.1%)	0.961
Segmental classification				
Proximal	178(61.0%)	130(60.5%)	48(62.3%)	0.879
Mid	220(75.3%)	165(76.7%)	55(71.4%)	0.439
Distal	34(11.6%)	23(10.7%)	11(14.3%)	0.525
Calcific arc grading				0.511
Grade 1,<90°	71(24.3%)	52(24.2%)	19(24.7%)	0.999
Grade 2,91°-180°	94(32.2%)	72(33.5%)	22(28.6%)	0.516
Grade 3181°-270°	81(27.7%)	55(25.6%)	26(33.8%)	0.219
Grade 4,>270°	46(15.8%)	36(16.7%)	10(13.0%)	0.552
Calcified nodule	129(44.2%)	90(41.9%)	39(50.7%)	0.231
Quantification of CAC				
Minimum lumen area, mm2	3.10(2.49,3.94)	3.25(2.57,4.13)	2.81(2.28,3.72)	0.027
Minimum lumen diameter, mm	1.79(1.58,2.07)	1.84(1.60,2.10)	1.73(1.54,1.97)	0.030
Min EEMA,mm2	10.98(9.51,13)	11.17(9.78,13.34)	10.85(8.97,12.57)	0.074
Min EEMD,mm	3.69 ± 0.47	3.71 ± 0.48	3.62 ± 0.44	0.107
Calcium_burden,%	71%(65%,77%)	71%(64%,77%)	72%(66%,77%)	0.534
Referencelumen area, mm2	7.76(6.35,9.32)	7.94(6.65,9.51)	7.12(5.95,8.54)	0.012
Reference lumen diameter, mm	2.88(2.60,3.14)	2.94(2.71,3.15)	2.8(2.52,3.04)	0.016
Reference EEMA,mm2	12.4(10.8,14.8)	12.9(11.2,15.1)	11.7(9.8,13.9)	0.003
Reference EEMD,mm	3.79(3.5,4.19)	3.88(3.58,4.22)	3.66(3.38,3.97)	0.002
Length of CAC,mm	16.3(8.0,25.5)	15.6(7.5,26.0)	18.0(9.1,25.0)	0.631
Remodeling index	0.89(0.77,1.02)	0.87(0.76,1.01)	0.92(0.82,1.08)	0.104
Lesions and PCI characteristics,n(%)				
Bifurcation	11(3.8%)	11(5.1%)	0	0.094
Chronic total occlusion	6(2.1%)	3(1.4%)	3(3.9%)	0.390
Intrastent restenosis	40(13.7%)	25(11.6%)	15(19.5%)	0.127
Treated with stent PCI	256(87.7%)	188(87.4%)	68(88.3%)	0.999
Single stent	75(25.7%)	51(23.7%)	24(31.2%)	0.258
Multiple stent	181(62.0%)	137(63.7%)	44(57.1%)	0.377
Treated with DEB PCI	35(12.0%)	26(12.1%)	9(11.7%)	0.999
Single DEB	30(10.3%)	26(12.1%)	4(5.2%)	0.136
Multiple DEB	5(1.7%)	0	5(6.5%)	0.001
Intraoperative use of GPI	47(16.1%)	37(17.2%)	10(13.0%)	0.494
Intraoperative use of IABP	10(3.4%)	7(3.3%)	3(3.9%)	0.999
Rotational atherectomy	23(7.9%)	17(7.9%)	6(7.8%)	0.999

CAC,coronary artery calcification; DEB,Drug-eluting balloon; EEMA, External Elastic Membrane Area; EEMD, External Elastic Membrane Diameter; GPI,Glycoprotein IIb/IIIa inhibitor; IABP,Intra-aortic balloon pump; IVUS, intravascular ultrasound; PCI,percutaneous coronary intervention.

#### 3.2.1 Calcium distribution and morphology

The distribution of calcification across the major coronary arteries (the left main, left anterior descending, left circumflex, and right coronary arteries) was comparable between the groups (all p > 0.05). Similarly, no significant differences were observed in the qualitative morphological characteristics of CAC. This included the prevalence of superficial calcium (97.7% vs. 98.7%, p = 0.939), deep calcium (20.9% vs. 22.1%, p = 0.961), segmental distribution (proximal, mid, distal), calcific arc grading (p = 0.511 for overall distribution), or the presence of calcified nodules (41.9% vs. 50.7%, p = 0.231).

#### 3.2.2 Quantitative CAC and vessel parameters

Quantitative IVUS analysis revealed significant alterations in vessel architecture associated with elevated Lp(a). Lesions in the high Lp(a) group presented a significantly smaller minimum lumen area (2.81 [2.28, 3.72] vs. 3.25 [2.57, 4.13] mm^2^, p = 0.027) and minimum lumen diameter (1.73 [1.54, 1.97] vs. 1.84 [1.60, 2.10] mm, p = 0.030). Furthermore, the reference segments adjacent to these lesions demonstrated consistently smaller dimensions in the high Lp(a) group, including the reference lumen area (7.12 [5.95,8.54] vs. 7.94 [6.65,9.51] mm^2^, p = 0.012), reference lumen diameter (2.80 [2.52,3.04] vs. 2.94 [2.71,3.15] mm, p = 0.016), reference external elastic membrane (EEM) area (11.7 [9.8,13.9] vs. 12.9 [11.2,15.1] mm^2^, p = 0.003), and reference EEM diameter (3.66 [3.38,3.97] vs. 3.88 [3.58,4.22] mm, p = 0.002). These findings collectively indicate a constrictive vascular remodeling pattern in patients with elevated Lp(a). In contrast, the calcium burden (71% [64%, 77%] vs. 72% [66%, 77%], p = 0.534) and calcification length (15.6 [7.5, 26.0] vs. 18.0 [9.1, 25.0] mm, p = 0.631) were comparable between the groups.

#### 3.2.3 Lesion and PCI procedural characteristics

The prevalence of complex lesion characteristics, including bifurcations (5.1% vs. 0%, p = 0.094), chronic total occlusions (1.4% vs. 3.9%, p = 0.390), and ISR (11.6% vs. 19.5%, p = 0.127), did not differ significantly between the low and high Lp(a) groups. The rates of stent implantation (87.4% vs. 88.3%, p = 0.999) and drug-eluting balloon (DEB) angioplasty (12.1% vs. 11.7%, p = 0.999) were also similar. However, the use of multiple DEBs was exclusively required and significantly more common in the high Lp(a) group (0% vs. 6.5%, p = 0.001). The utilization of adjunctive devices, such as rotational atherectomy (7.9% vs. 7.8%, p = 0.999) and intra-aortic balloon pumps (3.3% vs. 3.9%, p = 0.999), was comparable between the groups. Post-dilation balloons were routinely used following stent implantation to optimize expansion. However, newer calcium-modification techniques such as orbital atherectomy, intravascular lithotripsy, excimer laser coronary atherectomy (ELCA), or cutting/scoring balloons were not employed in this cohort during the study period.

### 3.3 Clinical outcomes

The median follow-up duration for the entire cohort was 17.2 months (IQR 14.4–21.3).

#### 3.3.1 Kaplan‒Meier analysis

**Fig. 3:**
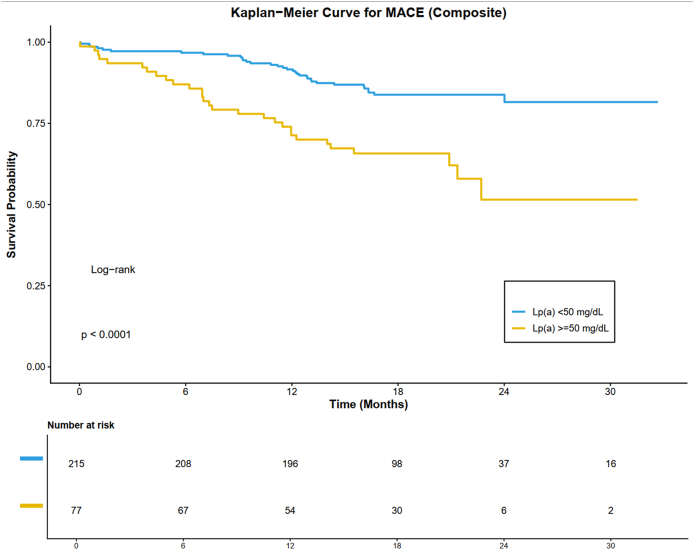
Kaplan-Meier Curves for the primary composite endpoint of MACEs. Lp(a), lipoprotein(a); MACE, major adverse cardiovascular events.

Elevated Lp(a) was associated with a significantly greater risk of the composite MACE endpoint (log-rank p < 0.001; Fig. 3). The risks of secondary endpoints, including cardiovascular death, stroke, and ISR, were also significantly elevated in the high Lp(a) group (all log-rank p < 0.05; Supplementary Fig. 3). In contrast, Lp(a) levels were not significantly associated with the risk of nonfatal MI, TVR, or heart failure (all log-rank p > 0.05; Supplementary Fig. 4). A subgroup analysis based on acute (ACS) versus nonacute clinical presentation revealed consistent trends for elevated MACE risk with high Lp(a) in both groups (Supplementary Table 4).

#### 3.3.2 Cox regression analysis

**Fig. 4:**
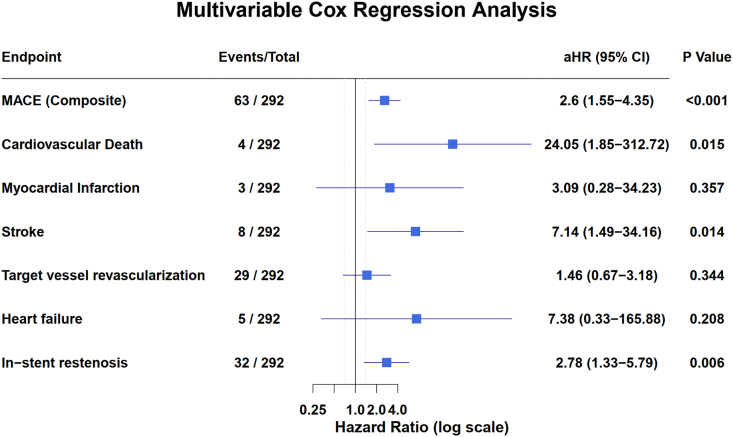
Association between Lp(a) levels and clinical outcomes in multivariable Cox regression analysis. The multivariable model included adjustments for age, gender, smoking, hypertension, hyperlipidemia, diabetes, history of myocardial infarction, history of percutaneous coronary intervention, and glomerular filtration rate. MACE, major adverse cardiovascular events.

Consistent with the results of the Kaplan‒Meier analysis, Cox regression models confirmed a significant association between elevated Lp(a) levels and adverse clinical outcomes (Table 3, Fig. 4). After multivariable adjustment for age, sex, smoking status, hypertension status, hyperlipidemia, diabetes status, prior MI, prior PCI, and GFR, elevated Lp(a) remained independently associated with a more than twofold increased risk of the primary composite MACE endpoint (adjusted hazard ratio [aHR] = 2.60, 95% CI: 1.55–4.35, p < 0.001).

**Table 3:** Incidence of MACE according to Lp(a) group.

Event,n(%)	Overall	Lp(a) < 50 mg/dL	Lp(a)≥50 mg/dL	aHR	P value
	(n = 292)	N = 215(73.6%)	N = 77(26.4%)	(95%CI)	
Primary Endpoint					
Composite MACEs	63(21.6%)	34(15.8%)	29(37.7%)	2.6 (1.55-4.35)	<0.001
Secondary Endpoints					
Cardiovascular death	4(1.4%)	1(0.5%)	3(3.9%)	24.05 (1.85-312.72)	0.015
Nonfatal MI	3(1.0%)	1(0.5%)	2(2.6%)	3.09 (0.28-34.23)	0.357
Stroke	8(2.7%)	3(1.4%)	5(6.5%)	7.14 (1.49-34.16)	0.014
TVR	29(9.9%)	18(8.4%)	11(14.3%)	1.46 (0.67-3.18)	0.344
Heart failure	5(1.7%)	3(1.4%)	2(2.6%)	7.38 (0.33-165.88)	0.208
In-stent restenosis	32(11.0%)	17(7.9%)	15(19.5%)	2.78 (1.33-5.79)	0.006

aHR,adjusted hazard ratio; CI,confidence interval; Lp(a), lipoprotein(a); MACEs, major adverse cardiovascular events; MI,myocardial infarction; TVR,Target vessel revascularization.

The adjusted Cox proportional hazards model included the following covariates:age, gender, smoking, hypertension, hyperlipidemia, diabetes, history of MI,history of PCI and glomerular filtration rate. The aHR for cardiovascular death is highly unstable due to the very low number of events (n = 4). This result should be interpreted with extreme caution and not considered as reliable evidence of an association.

Analysis of individual MACE components revealed a markedly greater risk of ischemic stroke in the high Lp(a) group (aHR = 7.14, 95% CI: 1.49–34.16, p = 0.014). The risk of ISR was also significantly elevated (aHR = 2.78, 95% CI: 1.33–5.79, p = 0.006). Although the incidence of cardiovascular death was numerically greater in the high Lp(a) group (3.9% vs. 0.5%), this estimate was highly unstable due to the low number of events (aHR = 24.05, 95% CI: 1.85–312.72, p = 0.015). The exceedingly wide confidence intervals indicate profound uncertainty in this estimate; thus, no reliable conclusions can be drawn regarding this specific endpoint. No significant associations were observed for nonfatal MI, TVR, or heart failure after multivariable adjustment.

#### 3.3.3 Sex-specific subgroup analysis

Subgroup analyses were performed to evaluate effect modification by sex. As detailed in Supplementary Table 3, the association between elevated Lp(a) and the primary MACE endpoint was more pronounced in men (aHR 3.26, 95% CI 1.87--5.70, p < 0.001) than in women (aHR 1.68, 95% CI 0.52--5.46, p = 0.389). However, the interaction effect between Lp(a) group and sex was not statistically significant (p for interaction = 0.312, Supplementary Fig. 5). Baseline characteristics stratified by sex and Lp(a) level are provided in Supplementary Table 1. Notably, the prevalence of hypertension was significantly greater in men with elevated Lp(a) than in men with low Lp(a) (76.3% vs. 59.6%, p = 0.035), a difference not observed in women. IVUS and procedural characteristics were largely consistent between Lp(a) groups within each sex stratum (Supplementary Table 2), with the constrictive remodeling phenotype (e.g., smaller reference vessel dimensions) being primarily evident among men in the high Lp(a) group. Although the point estimate suggested a stronger association in men, the formal test for interaction was not statistically significant, indicating that the effect of elevated Lp(a) on MACEs did not differ significantly by sex in this cohort.

## 4 Discussion

Elevated Lp(a) levels (≥50 mg/dL) are associated with a distinct systemic inflammatory-metabolic profile, a constrictive vascular remodeling phenotype, and a significantly increased risk of MACEs—primarily driven by ischemic stroke and ISR.

### 4.1 Study Position and unique contribution

Large-scale epidemiological studies (e.g., MESA, UK Biobank) have established Lp(a) as a causal risk factor for incident cardiovascular disease and CAC in general populations [8,17]. However, a critical translational gap exists in understanding its role in advanced, symptomatic atherosclerotic disease requiring intervention. Specifically, the associations of Lp(a) with in vivo lesion morphology, vascular remodeling, and procedure-related outcomes in this high-risk cohort remain poorly characterized.

Our study directly addresses this gap by focusing on a precisely defined, high-risk phenotype: patients with symptomatic coronary artery disease and intravascular ultrasound (IVUS)-confirmed, significant calcification in target lesions. Unlike computed tomography-based calcium scoring, which quantifies the global burden for risk prediction [18], IVUS provides high-resolution, lesion-specific characterization critical for guiding percutaneous coronary intervention [19]. This approach yields two key novel insights that extend beyond population-based epidemiology. First, we demonstrate for the first time that elevated Lp(a) is associated with an in vivo constrictive vascular remodeling pattern in advanced CAC, providing a direct anatomical correlation with clinical risk. Second, we identified a distinct, endpoint-specific risk profile, with elevated Lp(a) strongly linked to ischemic stroke and ISR but not myocardial infarction, highlighting a divergent athero-thrombotic phenotype.

Therefore, rather than merely replicating known epidemiological associations, our work translates systemic Lp(a) risk into actionable, lesion-specific pathophysiology for interventional cardiologists. It defines a high-risk subgroup characterized by a constrictive remodeling phenotype and a specific vulnerability to cerebrovascular events and stent failure, which may benefit from intensified surveillance and emerging targeted therapies.

### 4.2 Constrictive remodeling: A novel structural phenotype driven by Lp(a) pathobiology

IVUS-defined constrictive remodeling—manifested as a uniformly smaller reference lumen and vessel dimensions—provides a novel structural explanation for the elevated ischemic risk observed in patients with elevated Lp(a). We propose that this phenotype stems from pervasive Lp(a)-driven pathobiology [20]. In addition to being a carrier of cholesterol, Lp(a) is the principal transport vehicle for proinflammatory oxidized phospholipids (OxPLs). These OxPLs are potent inducers of arterial wall inflammation, eliciting a robust monocyte/macrophage response and promoting a profibrotic vascular milieu [21]. We hypothesize that this chronic, Lp(a)-mediated inflammatory state may drive pathological inward remodeling through two interrelated mechanisms: actively promoting perivascular and intimal fibrosis and impairing the compensatory outward expansion (Glagovian remodeling) that typically accompanies plaque growth [22]. This mechanistic link is supported by histological studies linking Lp(a) levels to fibrotic plaque characteristics [23]. The concomitant systemic inflammatory-metabolic profile (elevated hs-CRP and NT-proBNP) and the significantly greater burden of aortic valve calcification in the high Lp(a) group reinforce the concept of a widespread Lp(a)-mediated pathological process affecting both the coronary and valvular structures [24,25]. Thus, our IVUS approach reveals a distinct ‘constrictive’ architectural phenotype that may underlie the persistent risk in these patients and offers a structural endpoint for evaluating the efficacy of novel Lp(a)-lowering therapies.

### 4.3 Stroke and restenosis: defining a high-risk clinical phenotype and a dual-pathway hypothesis for stroke

Equally important is our identification of a divergent risk pattern, where elevated Lp(a) was strongly associated with ischemic stroke (aHR = 7.14) and in-stent restenosis (ISR, aHR = 2.78) but not with myocardial infarction [26]. This pattern likely reflects the potent thrombotic and impaired fibrinolytic effects of Lp(a), which might have a disproportionate impact on cerebrovascular territories and the stent‒vessel interface [21]. The strong link to ISR is congruent with Lp(a)'s role in inflammation and impaired healing, potentially exacerbating neointimal hyperplasia and thrombosis poststenting. Procedurally, the exclusive need for multiple drug-eluting balloons in the high Lp(a) group hints at greater underlying lesion complexity and resistance to conventional expansion, aligning with this increased restenosis risk. Large biobank studies, while powered for composite end points, often do not provide such granularity in prespecified, high-risk intervention subgroups. The strong link to stroke is supported by mechanistic studies implicating Lp(a) in carotid atherosclerosis and thromboembolism [23,27,28]. The magnitude of the association with ischemic stroke (aHR = 7.14) is particularly striking and warrants specific mechanistic consideration. We propose a dual-pathway hypothesis to explain this robust link: the direct thrombotic pathway: Lp(a) directly promotes cerebrovascular atherosclerosis, plaque instability, and thrombosis via its OxPL content and antifibrinolytic activity through structural homology with plasminogen. Indirect cardioembolic pathway: Notably, the prevalence of aortic valve calcification (AVC) was more than fourfold greater in the high Lp(a) group (23.4% vs. 5.6%). As Lp(a) is a causal driver of calcific aortic valve disease and AVC itself is an independent source of thromboembolism, elevated Lp(a) may concurrently increase stroke risk by creating a potential nidus for cardiogenic embolism [25]. This intriguing hypothesis—that Lp(a) elevates stroke risk both directly via cerebral vasculopathy and indirectly by promoting AVC—could explain the exceptionally high hazard ratio observed and merits validation in future studies with systematic echocardiographic screening and detailed stroke subtyping (e.g., TOAST classification) [8,29].

### 4.4 Clinical implications and future directions

The robust and independent association between elevated Lp(a) and adverse outcomes in our cohort underscores Lp(a) as a critical, yet largely unaddressed, residual risk factor in patients with advanced CAC—a risk evidently not mitigated by contemporary guideline-directed therapies [30]. Our findings provide a roadmap for translating this biomarker into actionable clinical practice and define a precise phenotype for future targeted therapies.

#### 4.4.1 Implications for clinical management

Our data advocate for a paradigm shift toward a more stratified management approach for patients with significant CAC.

● Systematic risk stratification: We propose the routine measurement of Lp(a) in all patients with moderate-to-severe CAC. An Lp(a) level ≥50 mg/dL identifies a “very-high-risk” phenotype that warrants distinct management considerations [10,31].
● Tailored Interventional Strategy: For patients with elevated Lp(a) levels undergoing PCI, preprocedural planning should anticipate more complex, calcific lesions. This may favor the proactive use of advanced calcium-modification techniques (e.g., rotational/orbital atherectomy, intravascular lithotripsy) to optimize stent expansion and apposition [32]. Furthermore, given the significantly elevated risk of in-stent restenosis, intensified post-PCI surveillance—potentially involving earlier anatomical or functional follow-up—is justified in this subgroup.
● Integrated Systemic Risk Management: The pronounced risk of ischemic stroke calls for heightened vigilance. In addition to ensuring optimal secondary cardiovascular prevention, collaborative management with neurology should be considered for comprehensive cerebrovascular risk assessment and prevention strategies [27,28].

#### 4.4.2 Future research directions

Our study also highlights critical avenues for future investigations.

● Elucidating the Role of Global Calcific Burden: Prospective studies that systematically integrate Lp(a) measurement with preprocedural coronary computed tomography angiography (to quantify the total coronary artery calcium score) are essential. Such designs will determine whether the excess risk we observed is specific to the confluence of high Lp(a) and severe focal calcification or is synergistic with a high global calcific burden.
● Validating the Dual-Pathway Stroke Hypothesis: The proposed mechanistic link between Lp(a), aortic valve calcification, and stroke requires formal validation. Future studies should incorporate systematic echocardiographic screening and detailed stroke subtyping (e.g., TOAST classification) to test this hypothesis.
● Defining the Target Population for Novel Therapies: Most importantly, our work defines an ideal candidate population for emerging Lp(a)-lowering therapies (e.g., pelacarsen and olpasiran) [33,34]. Patients matching our cohort definition—elevated Lp(a) (≥50 mg/dL) with imaging-confirmed moderate-to-severe coronary calcification—represent a high-priority group for enrollment in pivotal outcome trials. The finding that Lp(a) reduction in this group mitigates constrictive remodeling, stroke, and stent failure could offer profound clinical value and pave the way for a targeted therapeutic strategy.

### 4.5 Limitations

Our study has several limitations that merit consideration. First, and most pertinent to the interpretation of our findings, is the inherent selection bias associated with our IVUS-based design. While performing IVUS only on clinically indicated target lesions precisely defines our cohort as patients with symptomatic, locally advanced calcific disease requiring imaging-guided intervention—a major strength that enhances the clinical relevance of our results to interventional practice—it inherently precludes the assessment of the total coronary artery calcium burden. Consequently, a pivotal unanswered question emerges: is the excess risk associated with elevated Lp(a) specific to the confluence of high Lp(a) and severe focal calcification in a culprit lesion, or is it independent of, or synergistic with, the global calcific burden? Future prospective studies that systematically integrate Lp(a) measurement with either preprocedural coronary computed tomography angiography (to quantify the coronary artery calcium score) or routine multivessel intravascular imaging are essential to delineate their independent and synergistic prognostic contributions.

Second, the median follow-up duration of 17.2 months, while sufficient to capture procedure-related events such as in-stent restenosis and early ischemic strokes, may be inadequate for fully assessing the long-term risk of mortality and other hard cardiovascular endpoints attributable to elevated Lp(a). Extended follow-up is warranted.

Third, the retrospective, single-center design may be subject to residual confounding factors despite multivariable adjustments. Fourth, the sample size, although adequate for the primary composite endpoint, underpowered the analysis of infrequent individual events such as cardiovascular death; the exceedingly wide confidence intervals for this endpoint preclude definitive conclusions and highlight the need for caution in its interpretation. Fifth, although we performed sex-disaggregated analyses in accordance with SAGER guidelines, the sample size, particularly for women with elevated Lp(a) (n = 18), limited the statistical power to reliably detect effect modification by sex. The nonsignificant interaction term should therefore be interpreted with caution, and future studies with larger samples are needed to confirm or refute sex-based differences in the associations between Lp(a) levels and outcomes in patients with CAC.

## 5 Conclusion

In this well-characterized cohort of symptomatic patients with IVUS-confirmed CAC in target lesions, elevated Lp(a) (≥50 mg/dL) defines a high-risk subset marked by a constrictive vascular remodeling phenotype, a systemic inflammatory state, and a markedly increased burden of aortic valve calcification. Elevated Lp(a) independently predicts a substantially increased risk of MACEs, with ischemic stroke and in-stent restenosis as prominent drivers, highlighting a distinct athero-thrombotic profile. This evidence reinforces Lp(a) status as a key modifiable residual risk factor and supports the routine integration of Lp(a) screening into the management of patients with advanced coronary calcification for improved risk stratification and personalized care.

## CRediT authorship contribution statement

**Shaowu Xiao:** Writing – original draft, Visualization, Software, Methodology, Investigation, Formal analysis, Data curation, Conceptualization. **Mengya Zeng:** Writing – review & editing, Validation, Resources, Investigation. **Junru He:** Visualization, Validation, Investigation, Data curation. **Dabao Xiao:** Investigation, Data curation. **Yuewu Chen:** Writing – review & editing, Supervision, Project administration, Conceptualization.

## Ethics statement

The study was approved by the Ethics Committee of The Second Affiliated Hospital of Hainan Medical University (approval number LW2022035; Date: March 17, 2022) and was conducted in accordance with the Declaration of Helsinki, which waived the requirement for individual informed consent owing to the retrospective, anonymized nature of the analysis.

## Consent for publication

All the authors have read and approved the final submitted manuscript.

## Declaration of generative AI and AI-assisted technologies in the manuscript preparation process

During the preparation of this work, the authors used DeepSeek to refine the language and improve readability. After using this tool, the authors reviewed and edited the content as needed and take full responsibility for the content of the publication.

## Funding sources

This study was supported by the 10.13039/501100001809National Natural Science Foundation of China (82360063); the 10.13039/501100004761Natural Science Foundation of Hainan Province (High Level Talents Project) (821RC1127); the 10.13039/501100013142Key Research and Development Project of Hainan Province (Social Development) (ZDYF2022SHFZ070); and the Hainan Province Clinical Medical Center.

## Declaration of competing interest

None.

## Data Availability

Deidentified participant data and the study protocol will be made available upon reasonable request to the corresponding author, who is subject to a data use agreement and approval from the institutional ethics committee.

## References

[1] Villa-Bellosta R. (2021). Vascular calcification: key roles of phosphate and pyrophosphate. Int. J. Mol. Sci..

[2] Onnis C., Virmani R., Kawai K., Nardi V., Lerman A., Cademartiri F. (2024). Coronary artery calcification: current concepts and clinical implications. Circulation.

[3] Qiu Y., Hao W., Guo Y., Guo Q., Zhang Y., Liu X. (2024). The association of lipoprotein (a) with coronary artery calcification: a systematic review and meta-analysis. Atherosclerosis.

[4] Kronenberg F., Mora S., Stroes E.S.G., Ference B.A., Arsenault B.J., Berglund L. (2022). Lipoprotein(a) in atherosclerotic cardiovascular disease and aortic stenosis: a european atherosclerosis society consensus statement. Eur. Heart J..

[5] Nordestgaard B.G., Langsted A. (2024). Lipoprotein(a) and cardiovascular disease. Lancet Lond Engl.

[6] Kim B.J., Kang J. (2025). Association of lipoprotein(a) and coronary artery calcium with atherosclerotic cardiovascular disease. J Clin Lipidol.

[7] Bellomo T.R., Bramel E.E., Lee J., Urbut S., Flores A., Yu Z. (2025). Evaluation of lipoprotein(a) as a prognostic marker of extracoronary atherosclerotic vascular disease progression. Circulation.

[8] Averna M., Cefalù A.B. (2025). LP(a): the new marker of high cardiovascular risk. Nutr Metab Cardiovasc Dis NMCD.

[9] Delialis D., Manifava P., Giannakopoulou S.-P., Konstantaki C., Athanasopoulos S., Zervas G. (2025). Country-specific prevalence and clinical relevance of elevated Lp(a) as a risk enhancer in two Greek cohorts. J Clin Lipidol.

[10] Kronenberg F. (2022). Lipoprotein(a) measurement issues: are we making a mountain out of a molehill?. Atherosclerosis.

[11] Pu J., Mintz G.S., Biro S., Lee J.-B., Sum S.T., Madden S.P. (2014). Insights into echo-attenuated plaques, echolucent plaques, and plaques with spotty calcification: novel findings from comparisons among intravascular ultrasound, near-infrared spectroscopy, and pathological histology in 2,294 human coronary artery segments. J. Am. Coll. Cardiol..

[12] Markwerth P., Bajanowski T., Tzimas I., Dettmeyer R. (2021). Sudden cardiac death-update. Int. J. Leg. Med..

[13] Thygesen K., Alpert J.S., Jaffe A.S., Chaitman B.R., Bax J.J., Morrow D.A. (2018). Fourth universal definition of myocardial infarction (2018). Circulation.

[14] Hua X., Liu M., Wu S. (2023). Definition, prediction, prevention and management of patients with severe ischemic stroke and large infarction. Chin Med J (Engl).

[15] Metra M., Tomasoni D., Adamo M., Bayes-Genis A., Filippatos G., Abdelhamid M. (2023). Worsening of chronic heart failure: definition, epidemiology, management and prevention. A clinical consensus statement by the heart failure association of the european society of cardiology. Eur. J. Heart Fail..

[16] Giacoppo D., Mazzone P.M., Capodanno D. (2024). Current management of in-stent restenosis. J. Clin. Med..

[17] Lee H., Park K.S., Jeon Y.-J., Park E.J., Park S., Ann S.H. (2022). Lipoprotein(a) and subclinical coronary atherosclerosis in asymptomatic individuals. Atherosclerosis.

[18] Mokhtar J., Albaree M., Battistin V., Asbaita M., Akbarpoor F., Lakshmanan J. (2025). Inadequacy of coronary calcium scoring in evaluating coronary artery disease: a call to shifting to high-resolution CT coronary imaging. Int J Cardiol Cardiovasc Risk Prev.

[19] Kim Y., Bae S., Johnson T.W., Son N.-H., Sim D.S., Hong Y.J. (2022). Role of intravascular ultrasound-guided percutaneous coronary intervention in optimizing outcomes in acute myocardial infarction. J. Am. Heart Assoc..

[20] Tsimikas S., Brilakis E.S., Miller E.R., McConnell J.P., Lennon R.J., Kornman K.S. (2005). Oxidized phospholipids, Lp(a) lipoprotein, and coronary artery disease. N. Engl. J. Med..

[21] van der Valk F.M., Bekkering S., Kroon J., Yeang C., Van den Bossche J., van Buul J.D. (2016). Oxidized phospholipids on lipoprotein(a) elicit arterial wall inflammation and an inflammatory monocyte response in humans. Circulation.

[22] Byun Y.S., Lee J.-H., Arsenault B.J., Yang X., Bao W., DeMicco D. (2015). Relationship of oxidized phospholipids on apolipoprotein B-100 to cardiovascular outcomes in patients treated with intensive versus moderate atorvastatin therapy: the TNT trial. J. Am. Coll. Cardiol..

[23] Spence J.D. (2010). The role of lipoprotein(a) in the formation of arterial plaques, stenoses and occlusions. Can. J. Cardiol..

[24] Cardoso-Saldaña G., Fragoso J.M., Lale-Farjat S., Torres-Tamayo M., Posadas-Romero C., Vargas-Alarcón G. (2019). The rs10455872-G allele of the LPA gene is associated with high lipoprotein(a) levels and increased aortic valve calcium in a Mexican adult population. Genet. Mol. Biol..

[25] Khan M.I., Zahir R.S., Dominguez A.C., Romeo F.J. (2024). Role of lipoprotein (A) in aortic valve stenosis: novel disease mechanisms and emerging pharmacotherapeutic approaches. Int J Cardiol Heart Vasc.

[26] Emerging Risk Factors Collaboration, Erqou S., Kaptoge S., Perry P.L., Di Angelantonio E., Thompson A. (2009). Lipoprotein(a) concentration and the risk of coronary heart disease, stroke, and nonvascular mortality. JAMA.

[27] Wu C., Hou C., Zhao W., Li C., Chu X., Wu L. (2025). Lipoprotein(a), remote ischemic conditioning, and stroke recurrence in patients with symptomatic intracranial atherosclerotic stenosis. Neurother J Am Soc Exp Neurother.

[28] Lange K.S., Nave A.H., Liman T.G., Grittner U., Endres M., Ebinger M. (2017). Lipoprotein(a) levels and recurrent vascular events after first ischemic stroke. Stroke.

[29] Smolders B., Lemmens R., Thijs V. (2007). Lipoprotein (a) and stroke: a meta-analysis of observational studies. Stroke.

[30] Wang G., Xia M., Liang C., Pu F., Liu S., Jia D. (2024). Prognostic value of elevated lipoprotein (a) in patients with acute coronary syndromes: a systematic review and meta-analysis. Front. Cardiovasc. Med..

[31] Grundy S.M., Stone N.J., Bailey A.L., Beam C., Birtcher K.K., Blumenthal R.S. (2018). AHA/ACC/AACVPR/AAPA/ABC/ACPM/ADA/AGS/APhA/ASPC/NLA/PCNA guideline on the management of blood cholesterol: a report of the American college of cardiology/american heart association task force on clinical practice guidelines. Circulation.

[32] Demola P., Ristalli F., Hamiti B., Meucci F., Di Mario C., Mattesini A. (2020). New advances in the treatment of severe coronary artery calcifications. Cardiol. Clin..

[33] O'Donoghue M.L., Rosenson R.S., Gencer B., López J.A.G., Lepor N.E., Baum S.J. (2022). Small interfering RNA to reduce lipoprotein(a) in cardiovascular disease. N. Engl. J. Med..

[34] Alkhalil M. (2020). Lipoprotein(a) reduction in persons with cardiovascular disease. N. Engl. J. Med..

