# Supplementary material for: Elevated lipoprotein(a) and adverse outcomes in advanced coronary artery calcification: An intravascular ultrasound study
Source: Int J Cardiol Cardiovasc Risk Prev. 2026 Feb 20;29:200606. doi: 10.1016/j.ijcrp.2026.200606 (PMC12955567; doi:10.1016/j.ijcrp.2026.200606)
Supplement: Multimedia component 1 [file mmc1.pdf]

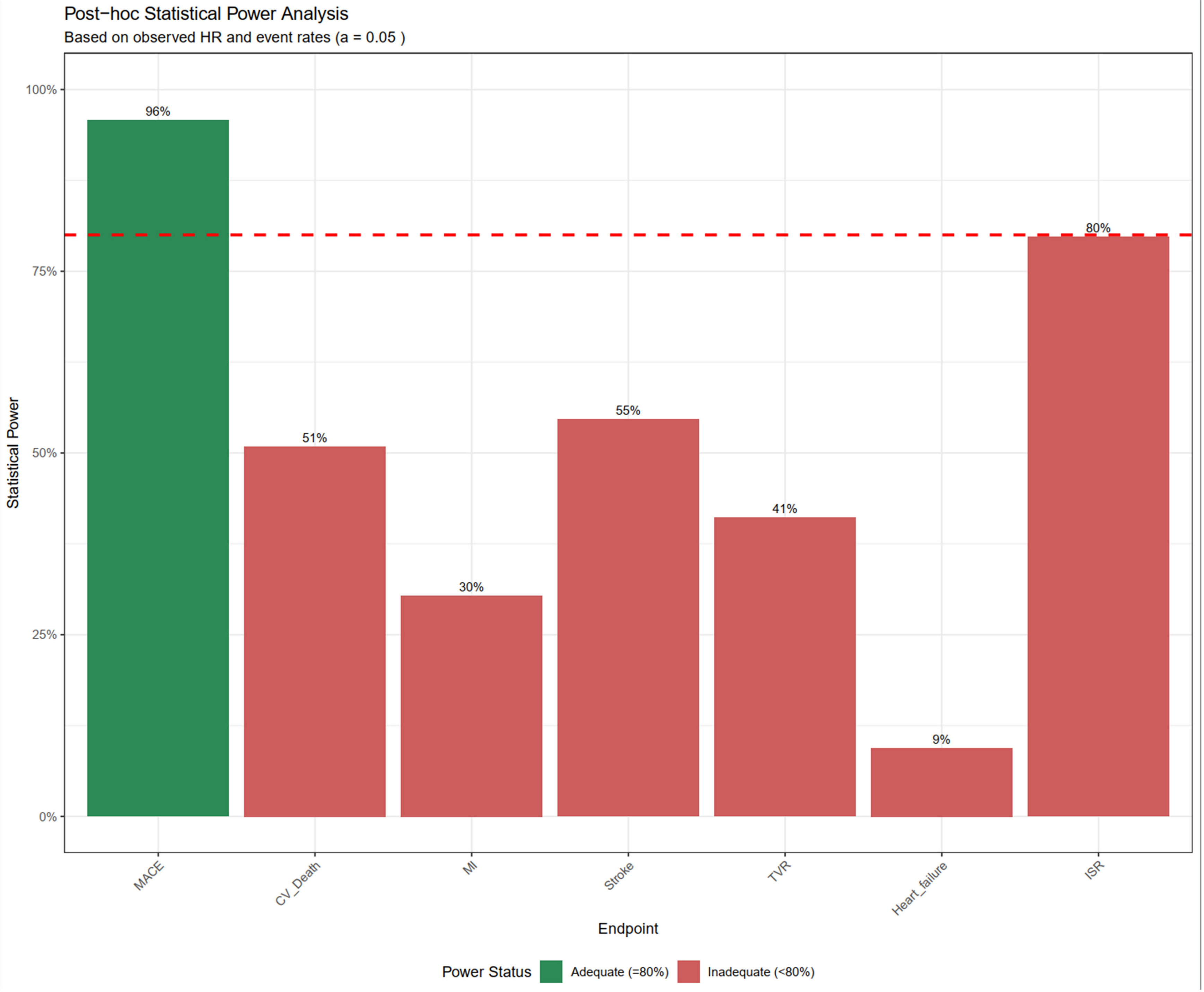

Supplementary Figure 1. A post-hoc power analysis.
CV, cardiovascular; ISR, in-stent restenosis; MACE, major adverse cardiovascular events; MI, myocardial infarction; TVR, target vessel revascularisation.

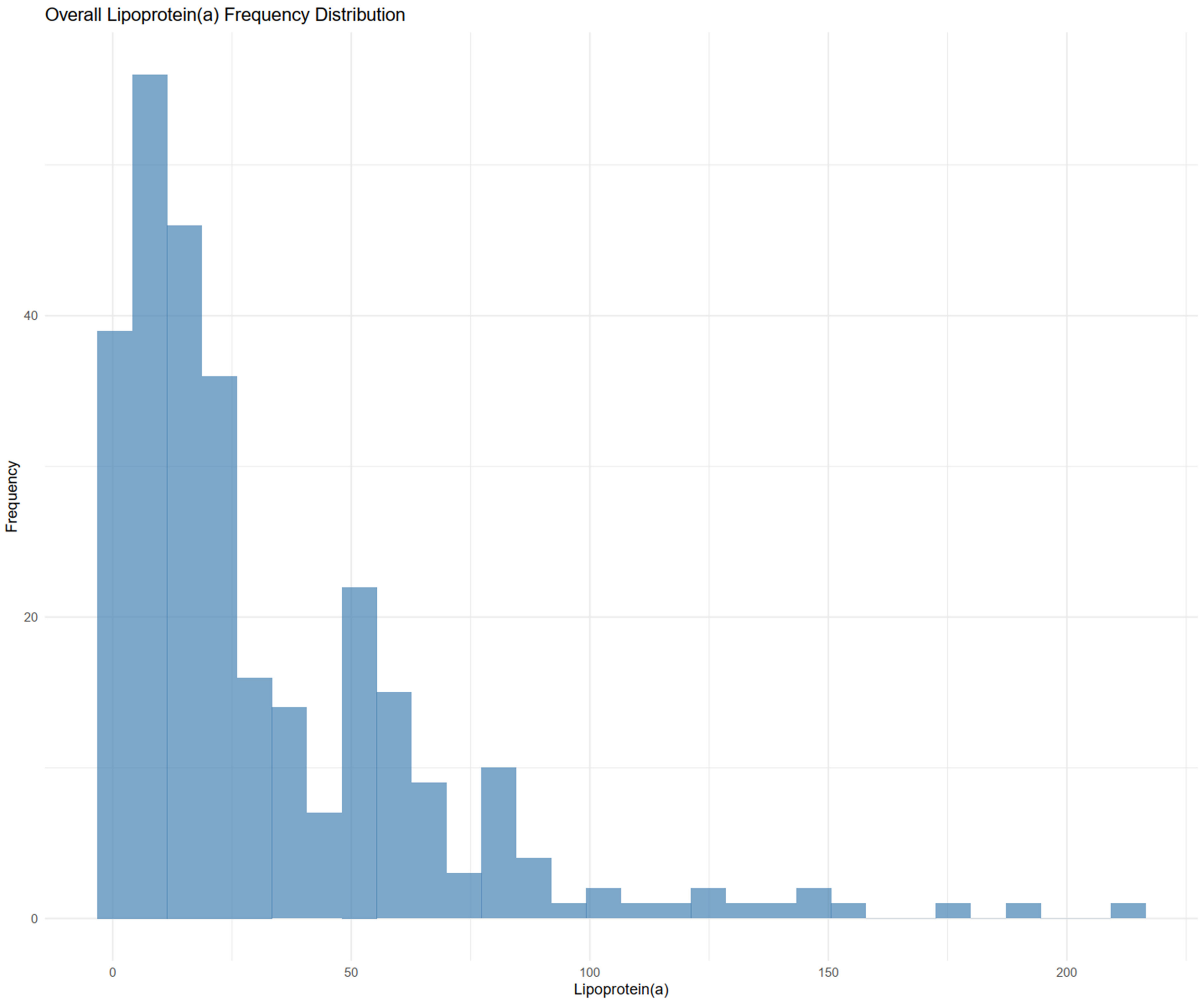

Supplementary Figure 2. Frequency distribution diagram of lipoprotein(a) concentration (mg/dL).

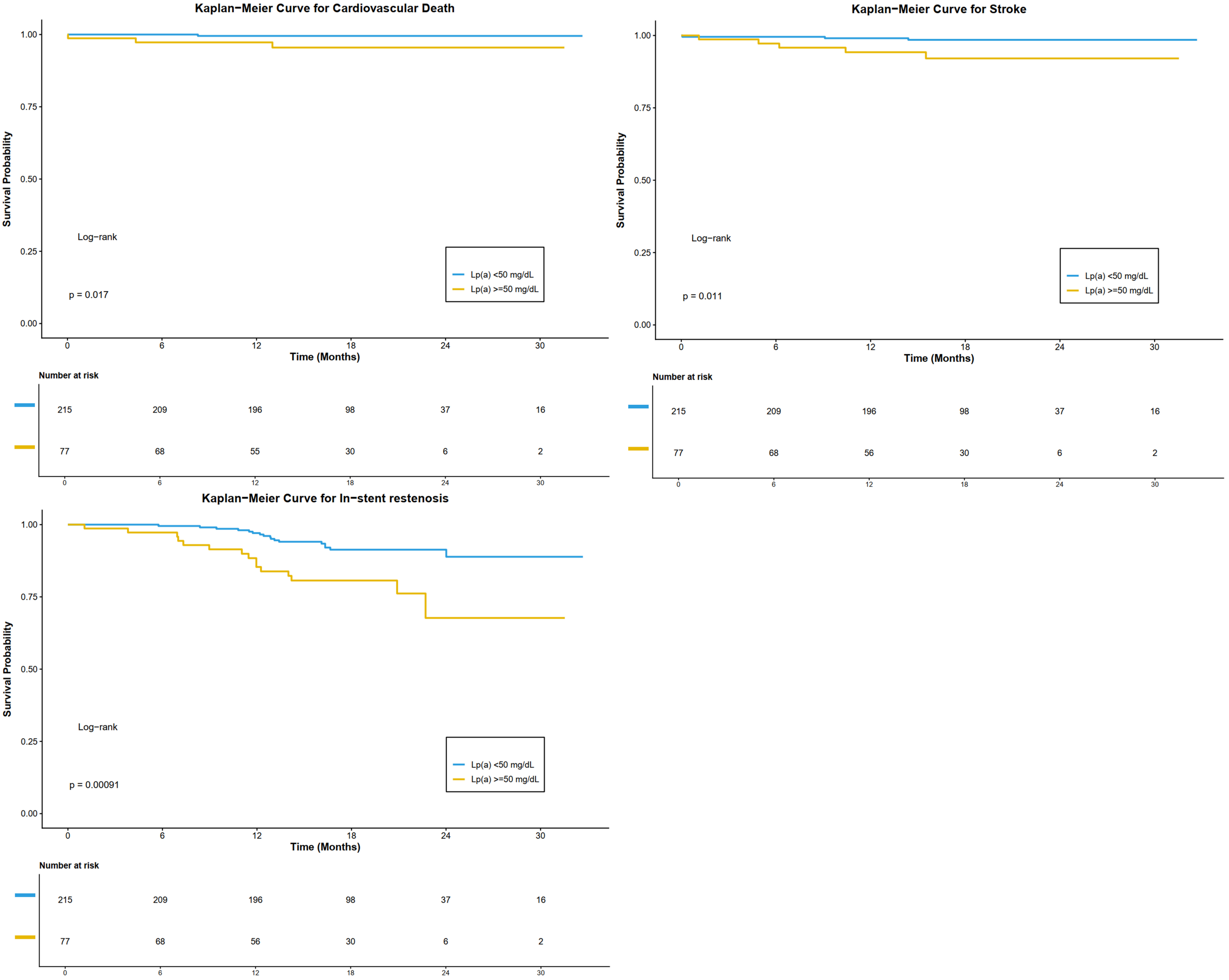

Supplementary Figure 3. Kaplan-Meier curves for significant secondary endpoints. Lp(a), lipoprotein(a).

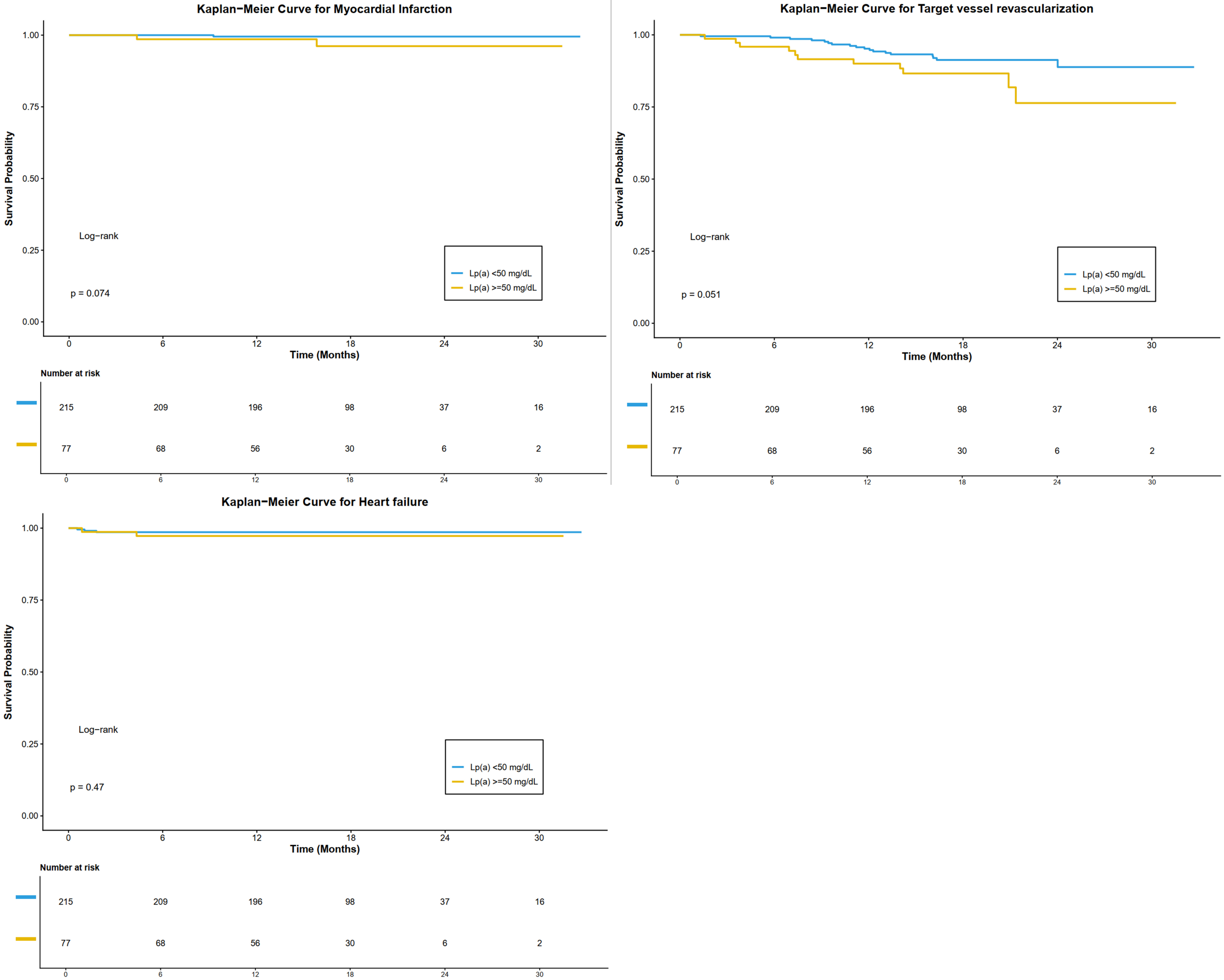

Supplementary Figure 4. Kaplan-Meier curves for non-significant secondary endpoints. Lp(a), lipoprotein(a).

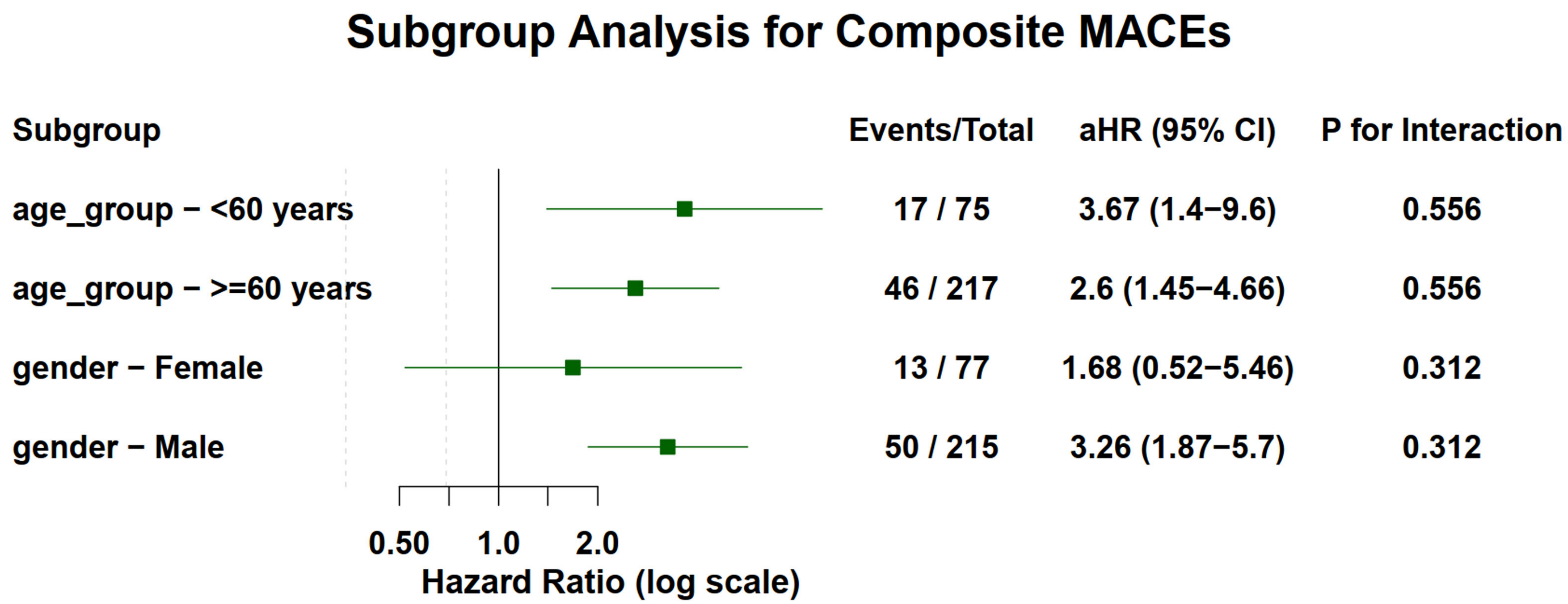

Supplementary Figure 5. Forest plot for age subgroup analysis.
MACE, major adverse cardiovascular events.
