# Supplementary material for: Elevated lipoprotein(a) and adverse outcomes in advanced coronary artery calcification: An intravascular ultrasound study
Source: Int J Cardiol Cardiovasc Risk Prev. 2026 Feb 20;29:200606. doi: 10.1016/j.ijcrp.2026.200606 (PMC12955567; doi:10.1016/j.ijcrp.2026.200606)
Supplement: Multimedia component 2 [file mmc2.docx]

| **Supplementary Table 1. Baseline Characteristics by Sex** | | | | | |
| --- | --- | --- | --- | --- | --- |
|  | Gender subgroup | Lp(a)< 50 mg/dL | Lp(a)≥50 mg/dL | P value | Interaction P Value |
|  |  | Female=77  Male=215 | Female=77  Male=215 |  |  |
| Patient demographics |  |  |  |  |  |
| Age,years | Female | 69.2±9.36 | 71.17±9.99 | 0.465 | 0.489 |
|  | Male | 65.11±11.16 | 64.83±9.65 | 0.857 |  |
| Body mass index,kg/m2 | Female | 23.3(20.3,26.4) | 23.0(21.5,24.4) | 0.952 | 0.98 |
|  | Male | 24.0(22.1,26.0) | 23.74(22.0,25.9) | 0.883 |  |
| SBP,mmHg | Female | 144(116,158) | 134.5(122,150) | 0.370 | 0.212 |
|  | Male | 133(120,153) | 143(119,159) | 0.217 |  |
| DBP,mmHg | Female | 78(72.5,87) | 74(69,87.75) | 0.596 | 0.894 |
|  | Male | 78(72,89) | 77(70,86.5) | 0.615 |  |
| Medical history,n(%) |  |  |  |  |  |
| Smoking | Female | 0 | 0 | NULL | 0.352 |
|  | Male | 62(39.7%) | 18(30.5%) | 0.275 |  |
| Hypertension | Female | 42(71.2%) | 11(61.1%) | 0.605 | 0.541 |
|  | Male | 93(59.6%) | 45(76.3%) | 0.035 |  |
| Dyslipidaemia | Female | 9(15.3%) | 3(16.7%) | 0.999 | 0.536 |
|  | Male | 36(23.1%) | 7(11.9%) | 0.100 |  |
| Diabetes mellitus | Female | 29(49.2%) | 7(38.9%) | 0.621 | 0.546 |
|  | Male | 52(33.3%) | 25(42.4%) | 0.283 |  |
| Chronic kidney disease | Female | 3(5.1%) | 1(5.6%) | 0.999 | 0.639 |
|  | Male | 8(5.1%) | 8(13.6%) | 0.070 |  |
| Prior myocardial infarction | Female | 9(15.3%) | 1(5.6%) | 0.502 | 0.556 |
|  | Male | 35(22.4%) | 13(22%) | 0.999 |  |
| Prior PCI | Female | 10(16.9%) | 1(5.6%) | 0.410 | 0.820 |
|  | Male | 51(32.7%) | 28(47.5%) | 0.065 |  |
| Laboratory |  |  |  |  |  |
| Glucose,mmol/L | Female | 6.12(5.44,8.45) | 6.83(5.3,10.67) | 0.524 | 0.600 |
|  | Male | 6.07(5.23,7.83) | 6.73(5.68,10.62) | 0.011 |  |
| Hemoglobin A1c,% | Female | 6.5(6.2,8.6) | 6.25(5.93,7.57) | 0.262 | 0.106 |
|  | Male | 6.2(5.9,7.12) | 6.5(6.05,7.4) | 0.234 |  |
| Serum creatinine,μmol/L | Female | 64(53.5,72.5) | 62(56.5,65.75) | 0.691 | 0.267 |
|  | Male | 85.5(74.8,97.8) | 96.0(77.5,118.0) | 0.025 |  |
| GFR,mL/min/1.73 m² | Female | 85.5(71.0,97.1) | 86.6(80.9,103.2) | 0.601 | 0.280 |
|  | Male | 84.0(68.8,96.3) | 75.2(58.2,92.7) | 0.050 |  |
| Triglycerides,mmol/L | Female | 1.66(1.09,2.09) | 1.69(1.29,2.21) | 0.531 | 0.805 |
|  | Male | 1.29(0.88,1.91) | 1.3(1.02,1.88) | 0.469 |  |
| Total cholesterol,mmol/L | Female | 4.65(3.76,5.21) | 4.56(4.02,5.76) | 0.448 | 0.164 |
|  | Male | 4.58(3.59,5.55) | 4.7(3.6,5.39) | 0.927 |  |
| LDL-C,mmol/L | Female | 2.8(2.06,3.3) | 2.57(2.27,3.24) | 0.553 | 0.650 |
|  | Male | 2.74(2.04,3.62) | 2.91(2.01,3.49) | 0.928 |  |
| HDL-C,mmol/L | Female | 1.07(0.9,1.29) | 1.06(0.98,1.35) | 0.732 | 0.694 |
|  | Male | 1.01(0.83,1.21) | 1.04(0.87,1.16) | 0.571 |  |
| Lp(a),mg/dL | Female | 13.5(6.4,23.3) | 67.2(54.8,82.9) | < 0.001 | 0.344 |
|  | Male | 12.8(5.5,22.6) | 62.9(55.3,85.5) | < 0.001 |  |
| CK-MB,U/L | Female | 12(9,18) | 12.5(9,25.47) | 0.682 | 0.199 |
|  | Male | 14(9,27) | 15(10,27.5) | 0.496 |  |
| Cardiac troponin I,ng/ml | Female | 0.01(0.01,1.31) | 0.01(0.01,1.67) | 0.422 | 0.823 |
|  | Male | 0.01(0.01,0.94) | 0.03(0.01,2.14) | 0.060 |  |
| NT-proBNP,pg/mL | Female | 249(83,1041) | 521(110,4499) | 0.276 | 0.158 |
|  | Male | 281(89,1154) | 963(159,2504) | 0.016 |  |
| White blood cell,×10⁹/L | Female | 7.24(5.67,10.11) | 7.2(5.53,8.36) | 0.777 | 0.766 |
|  | Male | 7.78(6.32,9.84) | 7.45(6.47,10.35) | 0.783 |  |
| Neutrophil,×10⁹/L | Female | 4.91(3.34,7.62) | 4.42(3.35,6.35) | 0.782 | 0.946 |
|  | Male | 4.67(3.78,6.72) | 5.03(4.05,8) | 0.248 |  |
| Monocyte,×10⁹/L | Female | 0.5(0.36,0.62) | 0.44(0.36,0.62) | 0.665 | 0.361 |
|  | Male | 0.5(0.43,0.69) | 0.55(0.43,0.74) | 0.439 |  |
| Platelet,×10⁹/L | Female | 239(204,277) | 236(205,290) | 0.563 | 0.398 |
|  | Male | 234(195,272) | 228(188,262) | 0.450 |  |
| hs-CRP,mg/L | Female | 3.96(1.29,9.62) | 4.56(1.82,19.14) | 0.370 | 0.260 |
|  | Male | 3.44(1.1,11.96) | 4.56(2.17,21.28) | 0.076 |  |
| Ejection fraction,% | Female | 62(55.5,65) | 56(44.8,67.5) | 0.433 | 0.768 |
|  | Male | 60(53,64) | 55(47,63) | 0.079 |  |
| Clinical presentation,n(%) |  |  |  |  |  |
| STEMI | Female | 5(8.5%) | 1(5.6%) | 0.999 | 0.545 |
|  | Male | 26(16.7%) | 8(13.6%) | 0.728 |  |
| NSTEMI | Female | 14(23.7%) | 5(27.8%) | 0.971 | 0.505 |
|  | Male | 22(14.1%) | 13(22%) | 0.231 |  |
| Unstable angina | Female | 27(45.8%) | 9(50%) | 0.964 | 0.598 |
|  | Male | 75(48.1%) | 31(52.5%) | 0.666 |  |
| Stable angina | Female | 13(22%) | 3(16.7%) | 0.873 | 0.613 |
|  | Male | 33(21.2%) | 7(11.9%) | 0.172 |  |
| Comorbidities,n(%) |  |  |  |  |  |
| Aortic valve calcification | Female | 2(3.4%) | 6(33.3%) | 0.001 | 0.568 |
|  | Male | 10(6.4%) | 12(20.3%) | 0.006 |  |
| NAVC | Female | 3(5.1%) | 1(5.6%) | 0.999 | 0.581 |
|  | Male | 6(3.8%) | 4(6.8%) | 0.583 |  |
| Aortic Stenosis | Female | 1(1.7%) | 0 | 0.999 | 0.588 |
|  | Male | 2(1.3%) | 1(1.7%) | 0.999 |  |
| Discharge Medications,n(%) |  |  |  |  |  |
| ACEI/ARB | Female | 35(59.3%) | 12(66.7%) | 0.777 | 0.581 |
|  | Male | 103(66%) | 36(61%) | 0.599 |  |
| Beta-blocker | Female | 37(62.7%) | 10(55.6%) | 0.788 | 0.653 |
|  | Male | 112(71.8%) | 47(79.7%) | 0.318 |  |
| statins | Female | 58(98.3%) | 18(100%) | 0.999 | 0.616 |
|  | Male | 153(98.1%) | 56(94.9%) | 0.428 |  |
| Dual antiplatelet therapy | Female | 53(89.8%) | 16(88.9%) | 0.999 | 0.623 |
|  | Male | 142(91%) | 57(96.6%) | 0.271 |  |
| Aspirin | Female | 54(91.5%) | 16(88.9%) | 0.999 | 0.602 |
|  | Male | 148(94.9%) | 57(96.6%) | 0.859 |  |
| Clopidogrel | Female | 38(64.4%) | 11(61.1%) | 0.999 | 0.572 |
|  | Male | 89(57.1%) | 37(62.7%) | 0.551 |  |
| Ticagrelor | Female | 21(35.6%) | 7(38.9%) | 0.999 | 0.585 |
|  | Male | 62(39.7%) | 22(37.3%) | 0.963 |  |
|  |  |  |  |  |  |
| ACE-I/ARBs,angiotensin-converting enzyme inhibitors/angiotensin receptor blockers;CK-MB,creatine kinase myocardial band;DBP,diastolic blood pressure;GFR,glomerular filtration rate;HDL-C,high-density lipoprotein;hs-CRP,high-sensitivity C-reactive protein;LDL-C,low-density lipoprotein;Lp(a),lipoprotein(a);NAVC,Non-aortic cardiac valve calcification;NSTEMI,non-ST segment elevation acute myocardial infarction;NT-proBNP,N-terminal pro-B type natriuretic peptide;PCI,percutaneous coronary intervention;SBP,systolic blood pressure;STEMI,ST segment elevation acute myocardial infarction. | | | | | |
|  |  |  |  |  |  |
|  |  |  |  |  |  |
|  |  |  |  |  |  |
|  |  |  |  |  |  |
|  |  |  |  |  |  |
|  |  |  |  |  |  |
|  |  |  |  |  |  |

| **Supplementary Table 2. IVUS and Procedural Characteristics of CAC by Sex** | | | | | |
| --- | --- | --- | --- | --- | --- |
|  | Gender subgroup | Lp(a)< 50 mg/dL | Lp(a)≥50 mg/dL | P value | p for interaction |
|  |  | Female=77,Male=215 | Female=77,Male=215 |  |  |
| Calcification Site,n(%) |  |  |  |  |  |
| Left main | Female | 15(25.4%) | 1(5.6%) | 0.173 | 0.590 |
|  | Male | 33(21.2%) | 11(18.6%) | 0.828 |  |
| Left anterior descending artery | Female | 45(76.3%) | 14(77.8%) | 0.999 | 0.585 |
|  | Male | 113(72.4%) | 43(72.9%) | 0.999 |  |
| Left circumflex artery | Female | 6(10.2%) | 0 | 0.365 | 0.513 |
|  | Male | 25(16%) | 7(11.9%) | 0.582 |  |
| Right coronary artery | Female | 11(18.6%) | 4(22.2%) | 0.999 | 0.594 |
|  | Male | 40(25.6%) | 15(25.4%) | 0.999 |  |
| CAC Characterization,n(%) |  |  |  |  |  |
| Depth-based classification |  |  |  |  |  |
| Superficial calcium | Female | 59(100%) | 18(100%) | 0.999 | 0.555 |
|  | Male | 151(96.8%) | 58(98.3%) | 0.892 |  |
| Deep calcium | Female | 10(16.9%) | 4(22.2%) | 0.874 | 0.594 |
|  | Male | 35(22.4%) | 13(22%) | 0.999 |  |
| Segmental classification |  |  |  |  |  |
| Proximal | Female | 37(62.7%) | 11(61.1%) | 0.999 | 0.584 |
|  | Male | 93(59.6%) | 37(62.7%) | 0.796 |  |
| Mid | Female | 44(74.6%) | 10(55.6%) | 0.212 | 0.542 |
|  | Male | 121(77.6%) | 45(76.3%) | 0.985 |  |
| Distal | Female | 5(8.5%) | 2(11.1%) | 0.999 | 0.614 |
|  | Male | 18(11.5%) | 9(15.3%) | 0.615 |  |
| Calcific arc grading |  |  |  |  |  |
| Grade 1,<90° | Female | 12(20.3.%) | 4(22.2%) | 0.999 | 0.590 |
|  | Male | 40(25.6%) | 15(25.4%) | 0.999 |  |
| Grade 2,91°-180° | Female | 21(35.6%) | 5(27.8%) | 0.742 | 0.598 |
|  | Male | 51(32.7%) | 17(28.8%) | 0.703 |  |
| Grade 3,181°-270° | Female | 16(27.1%) | 6(33.3%) | 0.831 | 0.576 |
|  | Male | 39(25%) | 20(33.9%) | 0.257 |  |
| Grade 4,>270° | Female | 10(16.9%) | 3(16.7%) | 0.999 | 0.597 |
|  | Male | 26(16.7%) | 7(11.9%) | 0.510 |  |
| Calcified nodule | Female | 20(33.9%) | 9(50%) | 0.339 | 0.658 |
|  | Male | 70(44.9%) | 30(50.8%) | 0.528 |  |
| Quantification of CAC |  |  |  |  |  |
| Minimum lumen area,mm2 | Female | 3.19(2.49,4.01) | 2.92(2.32,3.64) | 0.434 | 0.939 |
|  | Male | 3.27(2.59,4.17) | 2.79(2.27,3.74) | 0.036 |  |
| Minimum lumen diameter,mm | Female | 1.78(1.57,2.09) | 1.7(1.53,1.97) | 0.363 | 0.951 |
|  | Male | 1.85(1.63,2.11) | 1.73(1.54,1.96) | 0.040 |  |
| Min EEMA,mm2 | Female | 10.45(9.31,12.48) | 10.9(9.9,12.67) | 0.617 | 0.227 |
|  | Male | 11.5(10.07,13.76) | 10.78(8.79,12.47) | 0.022 |  |
| Min EEM diameter,mm | Female | 3.6±0.45 | 3.63±0.38 | 0.738 | 0.208 |
|  | Male | 3.76±0.48 | 3.61±0.45 | 0.042 |  |
| Calcium_burden,% | Female | 70%(61%,76%) | 74%(68%,76%) | 0.246 | 0.313 |
|  | Male | 72%(65%,77%) | 70%(66%,78%) | 0.968 |  |
| Reference lumen area,mm2 | Female | 7.65(6.24,8.69) | 7.25(5.97,9.05) | 0.833 | 0.276 |
|  | Male | 8.07(6.8,9.63) | 7.12(5.97,8.47) | 0.004 |  |
| Reference lumen diameter,mm | Female | 2.85(2.53,3.07) | 2.76(2.56,3.07) | 0.814 | 0.251 |
|  | Male | 2.98(2.73,3.19) | 2.81(2.52,3.02) | 0.007 |  |
| Reference EEMA,mm2 | Female | 12.06(10.13,14.41) | 11.84(10.17,13.65) | 0.822 | 0.181 |
|  | Male | 13.28(11.4,15.57) | 11.69(9.67,13.85) | 0.001 |  |
| Reference EEMD,mm | Female | 3.73(3.42,4.05) | 3.64(3.39,3.91) | 0.666 | 0.225 |
|  | Male | 3.95(3.63,4.25) | 3.66(3.37,4.02) | 0.001 |  |
| Length of CAC,mm | Female | 16(10.95,25.62) | 18.82(8.19,24.02) | 0.551 | 0.566 |
|  | Male | 15.31(6.61,26.19) | 18(10,25.5) | 0.376 |  |
| Remodeling index | Female | 0.88(0.76,1.03) | 0.95(0.83,1.09) | 0.431 | 0.658 |
|  | Male | 0.86(0.76,1) | 0.91(0.81,1.05) | 0.148 |  |
| Lesions and PCI characteristics,n(%) |  |  |  |  |  |
| Bifurcation | Female | 3(5.1%) | 0 | 0.779 | 0.590 |
|  | Male | 8(5.1%) | 0 | 0.171 |  |
| Chronic total occlusion | Female | 2(3.4%) | 0 | 0.999 | 0.567 |
|  | Male | 1(0.6%) | 3(5.1%) | 0.113 |  |
| Intrastent restenosis | Female | 5(8.5%) | 1(5.6%) | 0.999 | 0.710 |
|  | Male | 20(12.8%) | 14(23.7%) | 0.081 |  |
| Treated with stent PCI | Female | 52(88.1%) | 15(83.3%) | 0.897 | 0.589 |
|  | Male | 136(87.2%) | 53(89.8%) | 0.766 |  |
| Single stent | Female | 21(35.6%) | 7(38.9%) | 0.999 | 0.462 |
|  | Male | 30(19.2%) | 17(28.8%) | 0.183 |  |
| Multiple stent | Female | 31(52.5%) | 8(44.4%) | 0.740 | 0.491 |
|  | Male | 106(67.9%) | 36(61%) | 0.426 |  |
| Treated with DEB PCI | Female | 8(13.6%) | 0 | 0.227 | 0.586 |
|  | Male | 18(11.5%) | 9(15.3%) | 0.002 |  |
| Single DEB | Female | 8(13.6%) | 0 | 0.227 | 0.588 |
|  | Male | 18(11.5%) | 4(6.8%) | 0.438 |  |
| Multiple DEB | Female | 0 | 0 | 0.999 | 0.802 |
|  | Male | 0 | 5(8.5%) | 0.002 |  |
| Intraoperative use of GPI | Female | 11(18.6%) | 0 | 0.111 | 0.57 |
|  | Male | 26(16.7%) | 10(16.9%) | 0.999 |  |
| Intraoperative use of IABP | Female | 1(1.7%) | 1(5.6%) | 0.956 | 0.592 |
|  | Male | 6(3.8%) | 2(3.4%) | 0.999 |  |
| Rotational atherectomy | Female | 4(6.8%) | 2(11.1%) | 0.922 | 0.588 |
|  | Male | 13(8.3%) | 4(6.8%) | 0.926 |  |
|  |  |  |  |  |  |
| CAC,coronary artery calcification;DEB,Drug-eluting balloon;EEMA,External Elastic Membrane Area;EEMD,External Elastic Membrane Diameter;GPI,Glycoprotein IIb/IIIa inhibitor;IABP,Intra-aortic balloon pump;IVUS,intravascular ultrasound;PCI,percutaneous coronary intervention. | | | | | |
|  |  |  |  |  |  |
|  |  |  |  |  |  |
|  |  |  |  |  |  |
|  |  |  |  |  |  |
|  |  |  |  |  |  |

| **Supplementary Table 3. Incidence of MACEs by Lp(a)and Sex** | | | | | | |
| --- | --- | --- | --- | --- | --- | --- |
| **Event,n(%)** | Gender subgroup | Lp(a)< 50 mg/dL | Lp(a)≥50 mg/dL | aHR | P value | p for interaction |
|  |  | N =215(73.6%) | N = 77(26.4%) | (95%CI) |  |  |
| Primary Endpoint |  |  |  |  |  |  |
| Composite MACEs | Female | 9(15.3%) | 4(22.2%) | 1.68(0.52-5.46) | 0.389 | 0.312 |
|  | Male | 25(16.0%) | 25(42.4%) | 3.26(1.87-5.7) | <0.001 |  |
|  |  |  |  |  |  |  |
| aHR,adjusted hazard ratio;CI,confidence interval;Lp(a),lipoprotein(a);MACEs,major adverse cardiovascular events.The adjusted Cox proportional hazards model included the following covariates:age,gender,smoking, hypertension,hyperlipidemia,diabetes,pre-MI,pre-PCI and glomerular filtration rate. | | | | | | |
|  |  |  |  |  |  |  |
|  |  |  |  |  |  |  |
|  |  |  |  |  |  |  |
|  |  |  |  |  |  |  |
